# Students’ understanding of normal sexual behavior definition through evaluation and group discussion

**DOI:** 10.1192/j.eurpsy.2022.2082

**Published:** 2022-09-01

**Authors:** R. Shilko, S. Kumchenko

**Affiliations:** Lomonosov Moscow State University, Psychology, Moscow, Russian Federation

**Keywords:** group discussion, Definition, normal sexual behavior

## Abstract

**Introduction:**

The assumption that “normal” or typical sexual behavior exists is important for research and practice (van Lankveld, 2013). The definition of normal sexual behavior is multifaceted, that is why its adequate understanding by students in the course of sexuality requires some effort.

**Objectives:**

The focus of the research was the students’ understanding of complicated normal sexual behavior.

**Methods:**

24 students (20 women; aged from 20 to 37 with M=25.5 and SD=5.7) completed adapted and modified questionnaire (Kite, 1990) consisting 30 items concerning sexual behavior by deciding whether or not they consider each item as normal. Then the evaluations by each student on all items were generalized and discussed and summarized in this general form among all participants.

**Results:**

Only 2 items were considered by all students as a normal: concerning sex somewhere other than a bed and masturbation after marriage. Some items were evaluated as normal by the half of participants: fantasizing about a person other than one’s partner during sex; becoming aroused by peeping; dressing of the clothing of the other sex; having rape fantasies. Many items were characterized as normal by less or more than a half of the participants. Trying to answer the question of what elements of sexual behavior can be considered as normal, students aware the ambiguity of these assumptions and observe the variability in the opinions of other participants.

**Conclusions:**

Evaluation of different elements of sexual behavior and subsequent group discussion demonstrates for students some difficulties and uncertainty in defining normal sex behavior.

**Disclosure:**

No significant relationships.

